# Effects of *Eimeria acervulina* infection on the luminal and mucosal microbiota of the duodenum and jejunum in broiler chickens

**DOI:** 10.3389/fmicb.2023.1147579

**Published:** 2023-03-20

**Authors:** Philip M. Campos, Katarzyna B. Miska, Mark C. Jenkins, Xianghe Yan, Monika Proszkowiec-Weglarz

**Affiliations:** ^1^USDA-ARS Research Participation Program, Oak Ridge Institute for Science and Education (ORISE), Oak Ridge, TN, United States; ^2^USDA-ARS, NEA Bioinformatics, Statistics Group, Beltsville, MD, United States; ^3^Animal Biosciences and Biotechnology Laboratory, USDA-ARS, NEA, Beltsville Agricultural Research Center, Beltsville, MD, United States; ^4^Animal Parasitic Diseases Laboratory, USDA-ARS, NEA, Beltsville Agricultural Research Center, Beltsville, MD, United States; ^5^Environmental Microbial and Food Safety Laboratory, USDA-ARS, NEA, Beltsville Agricultural Research Center, Beltsville, MD, United States

**Keywords:** microbiota, Coccidiosis, broiler chickens, duodenum, jejunum, 16S

## Abstract

The intestinal disease coccidiosis, caused by *Eimeria* parasites, impacts nutrient absorption in broiler chickens, leading to weight gain depression and major losses in the poultry industry. To develop alternatives to antibiotics for treating infected chickens, the gut microbiota has been researched because of its association with health factors such as nutrient exchange, immune system modulation, digestive system physiology, and pathogen exclusion. The aim of this study was to determine the effect of *Eimeria acervulina* infection on the luminal and mucosal microbiota of both the duodenum (DuoL and DuoM) and jejunum (JejL and JejM) at multiple time points (days 3, 5, 7, 10, and 14) post-infection. 16S rRNA amplicon sequencing was utilized to characterize the microbiota and analyze differences in alpha and beta diversity between infected (IF) and control (C) birds at each time point. Alpha diversity differed between IF and C birds in DuoM and JejM microbiota. Combined with beta diversity results, DuoM microbiota appeared to be affected by infection in the longer-term, while JejM microbiota were affected in the shorter-term. Relative abundances of bacterial taxa known for short-chain fatty acid (SCFA) production, such as Lachnospiraceae, *Subdoligranulum*, and *Peptostreptococcaceae*, tended to be lower in IF birds for all four microbiota. Moreover, predicted functional abundances showed MetaCyc pathways related to SCFA production, especially butyrate, may be influenced by these differences in bacterial relative abundance. Our findings expand understanding of how *Eimeria* infection affects luminal and mucosal microbiota in the duodenum and jejunum, and further research on metagenomic function may provide insights on the degree of influence duodenal and jejunal bacteria have on chicken health.

## 1. Introduction

Coccidiosis, a disease caused by protozoal parasites of the genus *Eimeria*, leads to an estimated US $14 billion in annual losses worldwide by impacting poultry production (Blake et al., 2020). On poultry farms, *Eimeria acervulina*, *Eimeria maxima*, and *Eimeria tenella* are among the most prevalent species found to infect broiler chickens (Györke et al., 2013). Each *Eimeria* species tends to infect different sites in the gastrointestinal tract (GIT), with *E. acervulina* primarily infecting the duodenum and upper jejunum of the small intestine (Lillehoj and Trout, 1996). Nutrient absorption in chickens primarily occurs in the small intestine, underlining the importance of limiting the spread of *E. acervulina*. *E. acervulina* causes malabsorptive coccidiosis, a disease characterized by mucoid enteritis, where inflammation occurs in the intestinal tract and accumulation of mucus may lead to weight gain depression (Blake and Tomley, 2014). This weight gain depression, in addition to inefficient feed conversion and reduced egg production, contribute to poultry industry losses. In addition, *Eimeria* lesions can predispose chickens to infection by *Clostridium perfringens*, resulting in mortality by necrotic enteritis (Moore, 2016). Due to a transition away from antibiotics as a solution against disease, research has moved toward development of alternatives such as probiotics, prebiotics, herbal extracts, antioxidants, and essential oils (Bozkurt et al., 2014), requiring a greater understanding of gut microbiota bacteria and their functions.

Widespread use of 16S rRNA amplicon sequencing has revealed the importance of bacterial communities that comprise the gut microbiota within the chicken GIT. The gut microbiota is associated with factors influencing health, such as nutrient exchange, immune system modulation, digestive system physiology, and pathogen exclusion (Stanley et al., 2014; Clavijo and Flórez, 2018). Different regions of the chicken GIT contain different bacterial profiles. While the cecum contains the most absolute bacterial counts and is a highly diverse region, the duodenum, jejunum, and ileum tend to be less diverse and often consist of *Lactobacillus*, *Enterococcus, Turcibacter, Clostridium sensu stricto*, and bacteria from the Clostridium XI cluster (family Peptostreptococcaceae, genus *Romboutsia*) (Rychlik, 2020). Some species of *Lactobacillus* have been associated with weight gain in neonatal broiler chicks (Angelakis and Raoult, 2010), and *Lactobacillus* species are often included in probiotics tested for potential health benefits.

Although it is known duodenal and jejunal microbiota differ from cecal microbiota, less is known about the differences in luminal and mucosal microbiota within the duodenum and jejunum. Moreover, little is known on how infection may influence luminal and mucosal microbiota within these regions, as well as how the separate microbiota are affected over multiple time points. In a previous study on cecal luminal and mucosal microbiota, infection by *Eimeria tenella* appeared to lead to longer-term effects on luminal bacterial diversity, whereas mucosal bacterial diversity was affected only at the peak of infection (Campos et al., 2022b). The aim of this study was to determine the effect of *E. acervulina* on the luminal and mucosal microbiota of both the duodenum and jejunum at multiple time points during infection. Greater understanding of how luminal and mucosal microbiota in the duodenum and jejunum are affected during *E. acervulina* infection may provide better insight on how bacteria in the small intestine are affected by *E. acervulina* and how these effects could impact poultry health.

## 2. Materials and methods

### 2.1. Animal care and tissue sampling

All animal care procedures were approved by the Institutional Animal Care and Use Committee (IACUC, protocol #18-025) of the Beltsville Agricultural Research Center (BARC). Ross 708 male broilers (288 birds, 1 d of age) were obtained from Longnecker’s Hatchery (Elizabethtown, PA) and placed into 1.00 m^2^ open-top wire brooder pens (25 chicks per pen). Birds were moved at 19 days of age into 72 finisher units (Alternative Designs, Siloam Springs, AR) with 4 birds per pen. A corn-soybean-based diet (approximately 24% crude protein in crumble format) and water were provided to chicks *ad libitum* for the duration of the study. One-half of the birds (144 birds) were infected (IF) with 1 × 10^5^ *E. acervulina* oocysts (USDA #12 isolate) in a volume of 1.0 mL per bird by oral gavage at 21 days of age, while the remaining 144 birds were sham-infected with water (control [C]). The resulting 36 pens of C birds and 36 pens of IF birds were placed in separate areas of the facility to prevent cross-transmission.

Of the 4 birds per pen, 1 bird closest to the pen’s average weight (to prevent sampling of birds with outlier weights) was euthanatized at day 0, 3, 5, 7, 10, and 14 post-infection (PI) *via* cervical dislocation. There were 6 pens (*n* = 6 replicates per treatment) for each time point, resulting in 36 C birds and 36 IF birds euthanatized. Infection was evaluated in another study, with plasma carotenoid concentrations being significantly lower in IF birds on days 5 and 7 post-infection, body weight being significantly lower in IF birds overall, and crypt depths being increased in IF birds, indicating an observable *E. acervulina* infection (Cloft et al., 2023). For luminal and mucosal microbiota sampling, a total of 48 of the 72 birds were randomly chosen as follows: 25 IF birds consisting of 5 replicates per day PI (3, 5, 7, 10, 14) and 23 C birds consisting of 5 replicates from d 0; 4 from each of d 3, 7, and 14; and 3 from each of d 5 and 10. The duodenal loop and a 5 inch section of the jejunum proximal to the duodenal-jejunal junction were dissected, and the duodenal contents (DuoL), duodenal epithelial scrapings (DuoM), jejunal contents (JejL), and jejunal epithelial scrapings (JejM) were collected. Isolated specimens were snap frozen in liquid nitrogen and stored at −80°C until bacterial DNA isolation.

### 2.2. Library preparation and sequencing

DNA extraction, library preparation, and sequencing were performed as described in Campos et al. (2022b), utilizing a DNeasy PowerSoil kit (Qiagen, Valencia, CA), PCR primers targeting the V3-V4 region of the 16S rRNA gene, and the Illumina MiSeq platform (Illumina, Inc., San Diego, CA), respectively. The 16S rRNA gene sequences determined in this study were deposited in the NCBI Sequence Read Archive database (SRA accession no. PRJNA892608).

### 2.3. Bioinformatics and data analysis

The bioinformatics platform Quantitative Insights Into Microbial Ecology 2 (QIIME 2, RRID:SCR_008249) (Bolyen et al., 2019) version 2021.4 was used to analyze the DuoL, DuoM, JejL, and JejM microbiota. Demultiplexed, paired-end sequence data were denoised with DADA2 (Callahan et al., 2016) *via* the q2-dada2 plugin, using a quality cutoff of 30 to determine truncation settings. A feature classifier was created *via* q2-feature-classifier (Bokulich et al., 2018) fit-classifier-naive-bayes using SILVA (RRID:SCR_006423) version 138 99% operational taxonomic unit reference sequences and taxonomy (Quast et al., 2012). Reads were extracted from reference sequences using the V3-V4 region forward and reverse primers prior to this step. The SILVA database was chosen over the commonly used Greengenes database due to previous studies demonstrating SILVA’s larger database size and more recent updates to taxonomy, potentially improving interpretation of results (Balvočiūtė and Huson, 2017; Campos et al., 2022a). The SILVA reference sequences and taxonomy files were pre-formatted using RESCRIPt (obtained at https://docs.qiime2.org/2022.2/data-resources/), a process used to reduce inconsistencies and improve processing by removing duplicate sequences that are assigned different taxonomies (Robeson et al., 2021). Amplicon sequence variants (ASVs) from DADA2 were assigned taxonomy *via* the q2-feature-classifier classify-sklearn naïve Bayes taxonomy classifier. Mitochondria and chloroplasts were filtered and excluded from the feature table. All ASVs were aligned with MAFFT (Katoh et al., 2002) *via* q2-alignment and used to construct a phylogeny with fasttree2 (Price et al., 2010) *via* q2-phylogeny. Rarefaction, or subsampling without replacement, was performed by considering diversity captured at various sampling depths (shown by alpha rarefaction plots produced *via* q2-diversity) and the number of samples that would be retained in the subset. The following sampling depths were applied for alpha and beta diversity analyses *via* q2-diversity: 20,917 for DuoL, 30,092 for DuoM, 16,730 for JejL, and 10,255 for JejM (Table 1).

**Table 1:** Sequencing summary of the four microbiota datasets processed in QIIME 2.

	DuoL	DuoM	JejL	JejM
Number of samples	48	48	48	48
Raw reads	7,054,436	9,619,588	5,843,163	3,491,242
Reads after QC^1^	5,094,132	5,719,142	4,130,104	2,157,364
Reads after filtering^2^	3,248,826	4,353,866	2,490,573	1,276,891
Reads per sample (range)	6–206,766	5,794–472,561	370–163,213	505–70,854
Mean reads per sample	67,684	90,706	51,887	26,602
Sampling depth^3^	20,917	30,092	16,730	10,255
Total number of ASVs^4^	1,234	4,524	1,044	3,050
ASV read length (range)	290–524	261–461	290–450	269–469
Mean ASV read length	433	287	435	385

^1^QC = quality control (via DADA2), ^2^Filtering involved exclusion of mitochondria and chloroplasts, ^3^Following rarefaction for diversity analyses, ^4^ASVs, amplicon sequence variants.

The alpha diversity metrics analyzed included the Shannon diversity index, observed features (ASVs), Faith’s phylogenetic diversity (Faith PD) (Faith, 1992), and evenness. Alpha diversity metrics were used to measure species richness and/or evenness within one sample, and the non-parametric Kruskal-Wallis test was used to analyze differences in alpha diversity between treatment groups. Observed features and Faith PD are both metrics of richness, however, Faith PD considers the phylogenetic differences in ASVs (Faith, 1992). Beta diversity was measured using UniFrac distance metrics, which incorporate phylogenetic distances (Lozupone and Knight, 2005). Unweighted UniFrac considers the presence and absence of ASVs in samples, while weighted UniFrac considers the abundance of ASVs (Lozupone et al., 2007). To test for significance in UniFrac distances, the non-parametric permutational analysis of variance (PERMANOVA) test was used. Principal coordinates analysis (PCoA) was used to visualize these distances between treatment groups as well as to visualize clustering of samples with similar microbiota. Visualizations for alpha diversity metrics and beta diversity PCoA were produced in R 4.0.3 (R Core Team, 2020) using the packages QIIME2R 0.99.35 (Bisanz, 2018) to import QIIME 2 PCoA results and tidyverse 1.3.0 (RRID:SCR_019186) (Wickham et al., 2019) for data wrangling with dplyr (RRID:SCR_016708) and graph production with ggplot2 (RRID:SCR_014601).

The linear discriminant analysis effect size (LEfSe, RRID:SCR_014609) algorithm (Segata et al., 2011) was used to identify features or taxa with significant differential abundance between C and IF birds. Phylogenetic Investigation of Communities by Reconstruction of Unobserved States 2 (PICRUSt2, RRID:SCR_022647) software was used to predict functional abundances based on marker gene sequences (Douglas et al., 2020). The MetaCyc (RRID:SCR_007778) Metabolic Pathways Database (Caspi et al., 2020) was used to produce functional abundance data, and the data were analyzed and visualized using STAMP 2.1.3 (RRID:SCR_018887) (Parks et al., 2014) to determine biological relevance of features.

## 3. Results

### 3.1. Microbiota profiles

**Figure 1:**
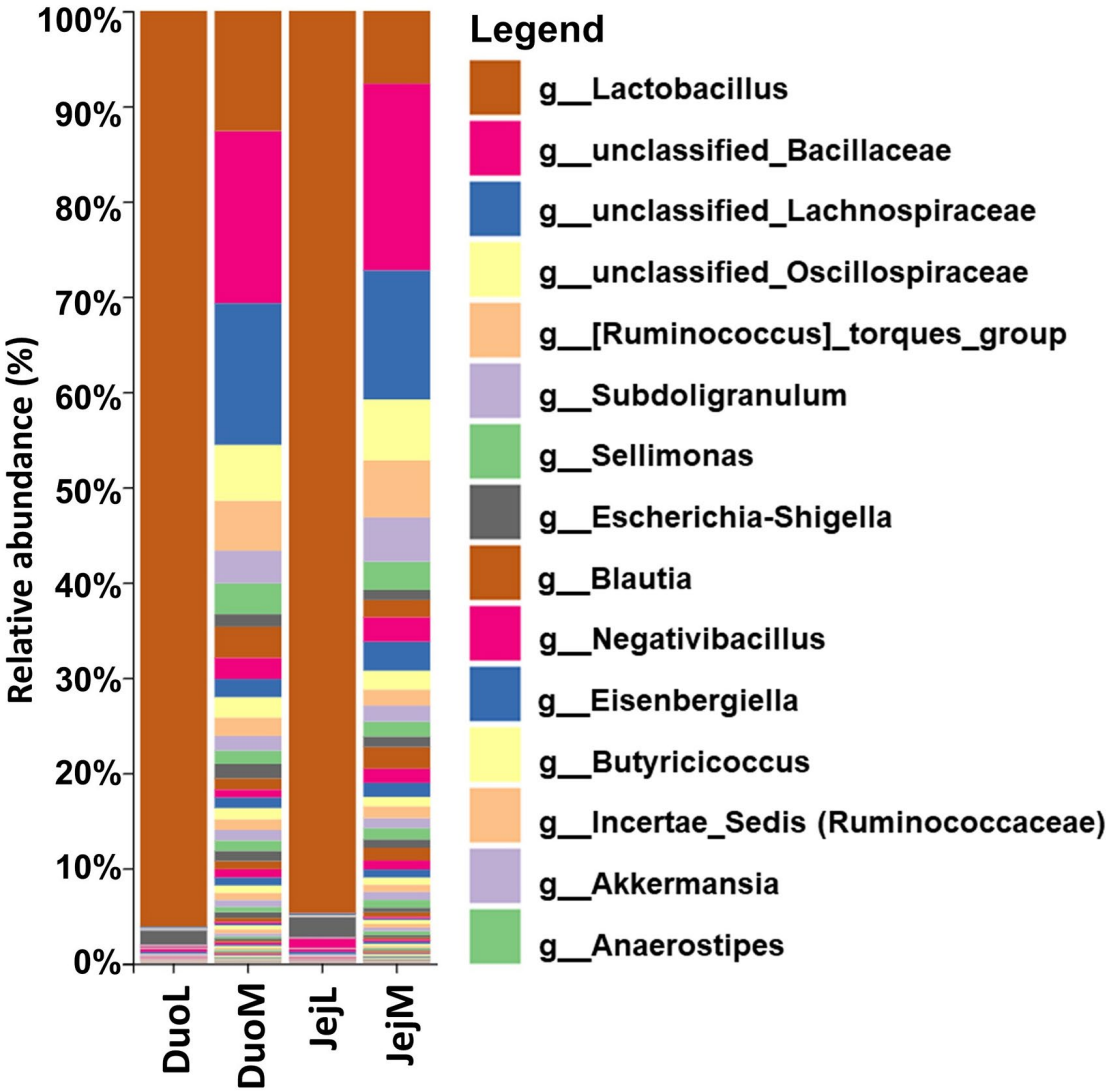
Relative abundances of bacterial taxa at the genus level in duodenal luminal (DuoL), duodenal mucosal (DuoM), jejunal luminal (JejL), and jejunal mucosal (JejM) microbiota (*n* = 48 samples for each column). The 15 most abundant taxa overall are indicated in the legend.

Sequencing summaries for the four microbiota datasets (DuoL, DuoM, JejL, and JejM) are presented in Table 1. The most abundant bacterial genera in DuoL microbiota were *Lactobacillus*, *Escherichia-Shigella*, unclassified Peptostreptococcaceae, *Streptococcus*, and unclassified Lachnospiraceae (Figure 1). *Lactobacillus* was dominant, comprising over 90% of the microbiota in 35 of the 48 samples, while only in 3 samples did the percentage of *Lactobacillus* fall below 50%. The majority of lactobacilli were unclassified at species level, and while 16S identification is not reliable at species level, the classifier reported that *Lactobacillus ingluviei* and *Lactobacillus phage* were the second and third most abundant at species level, respectively.

In DuoM microbiota, unclassified Bacillaceae, unclassified Lachnospiraceae, *Lactobacillus*, unclassified Oscillospiraceae, and [*Ruminococcus*] torques group were the most abundant bacterial genera (Figure 1). These five taxa were also the most abundant bacterial genera in JejM microbiota (Figure 1). Compared to DuoL microbiota, *Lactobacillus* (mostly unclassified or *L. ingluviei*) was not as dominant in DuoM microbiota, making up over 50% of the microbiota in only 2 samples. In JejL microbiota, the most abundant bacterial genera were *Lactobacillus*, *Escherichia-Shigella*, *Romboutsia*, *Streptococcus*, and unclassified Peptostreptococcaceae (Figure 1). *Lactobacillus* dominance was similar to that of DuoL microbiota, where 32 samples contained over 90% *Lactobacillus*.

### 3.2. Alpha diversity

**Figure 2:**
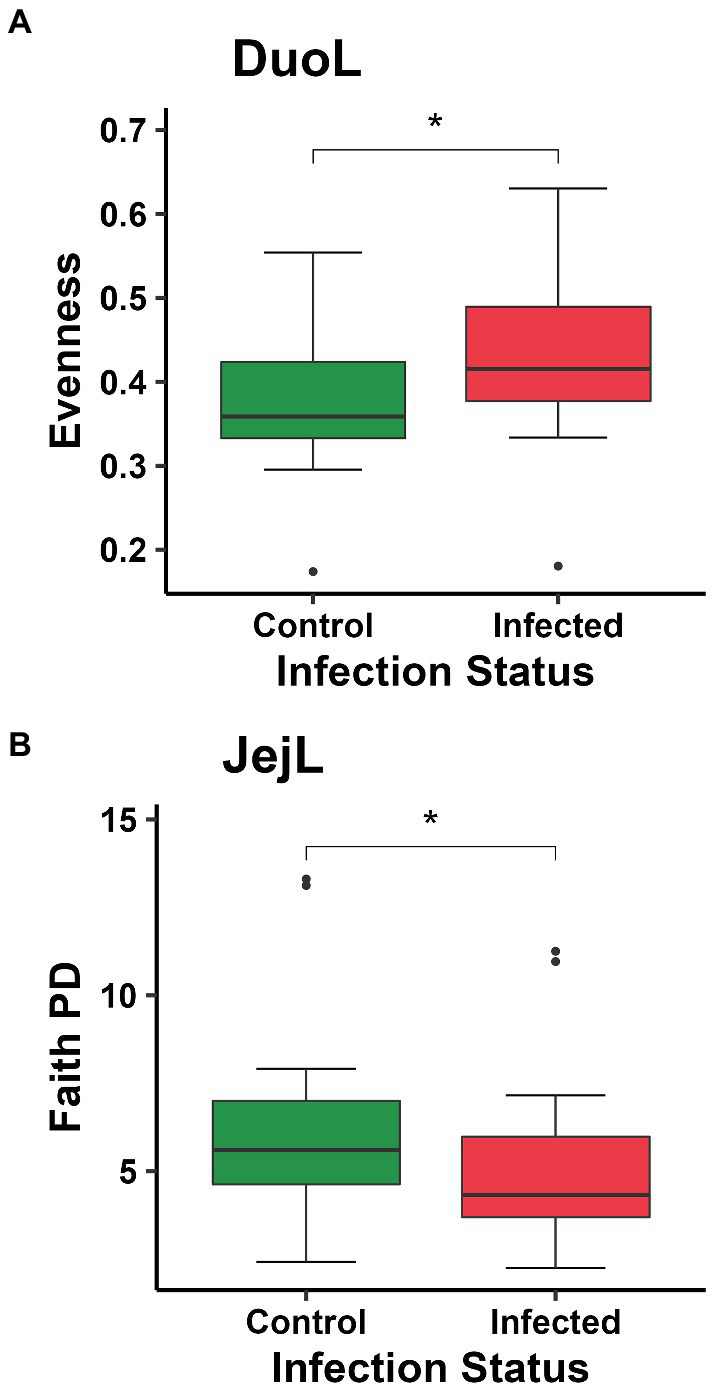
Comparisons of: **(A)** evenness between control and *Eimeria acervulina*-infected birds in duodenal luminal (DuoL) microbiota; **(B)** Faith’s phylogenetic diversity (Faith PD, richness) between control and *Eimeria acervulina*-infected birds in jejunal luminal (JejL) microbiota. Stars (*) denote statistically significant (*p* < 0.05) differences.

**Figure 3:**
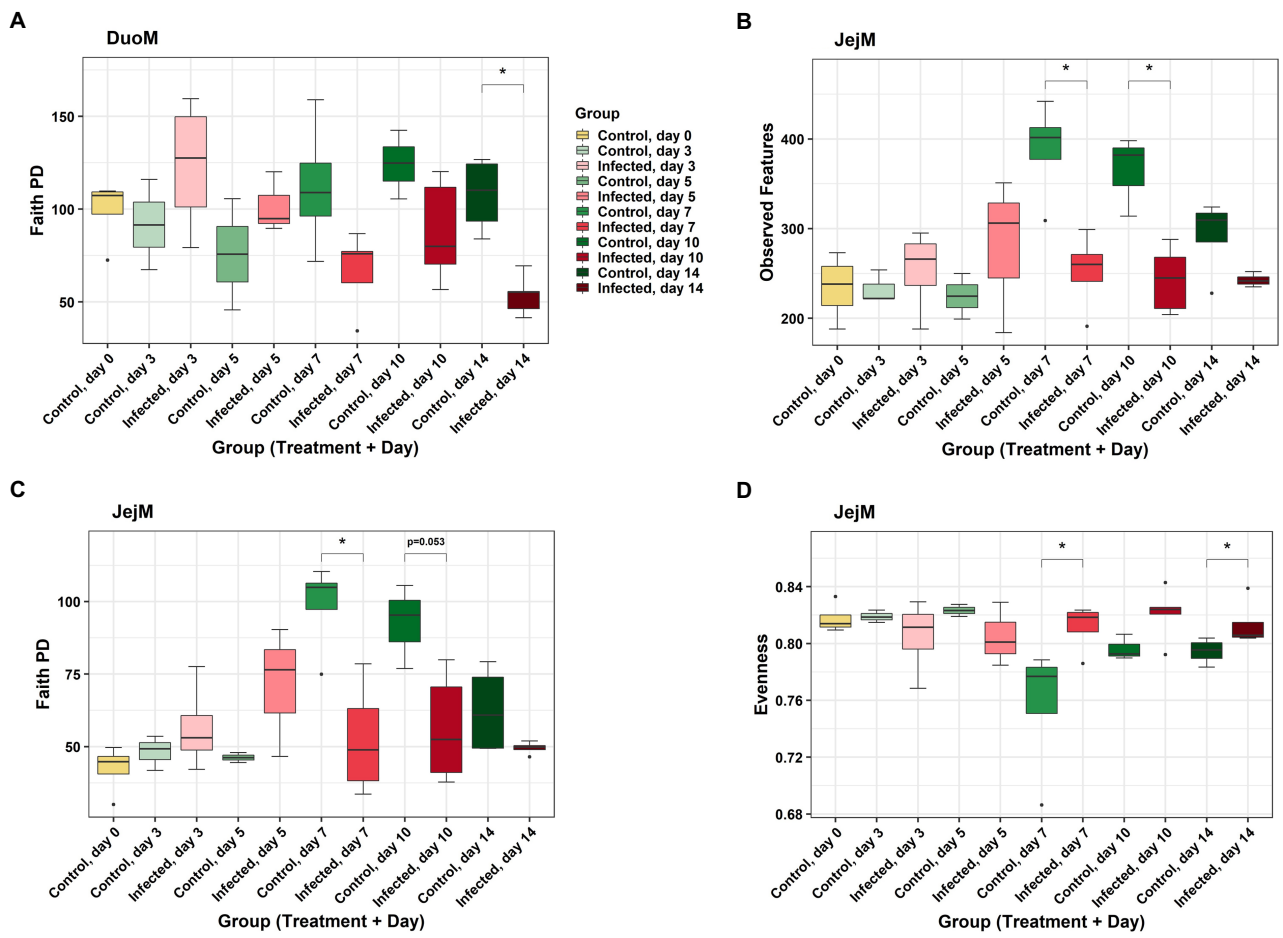
Comparisons of alpha diversity metrics between *Eimeria acervulina*-infected birds and control birds at multiple time points in duodenal mucosal (DuoM) microbiota, **(A)** Faith’s phylogenetic diversity (Faith PD, richness), and in jejunal mucosal (JejM) microbiota: **(B)** number of observed features (ASVs), **(C)** Faith’s phylogenetic diversity (richness), and **(D)** evenness. Stars (*) denote statistically significant (*p* < 0.05) differences between groups.

In DuoL microbiota, evenness was higher in *E. acervulina*-infected birds compared to control birds (Kruskal-Wallis, *H* = 4.28, *p* = 0.04, Figure 2A), however, there were no significant differences at a particular day post-infection based on the group (infection status and day post-infection) analysis (data not shown). There were no significant differences (*p* > 0.05) based on group, infection status, or time for Shannon diversity, observed features, and Faith PD. In DuoM microbiota, Faith PD significantly differed based on group (*H* = 21.04, *p* = 0.02, Figure 3A), with infected birds having lower Faith PD on day 14. There were no significant differences (*p* > 0.05) based on group, or infection status and time individually, in the remaining three alpha diversity metrics (data not shown).

In JejL microbiota, Faith PD was lower in infected birds compared to control birds (*H* = 4.23, *p* = 0.04, Figure 2B), however, there were no significant differences at a particular time (data not shown). There were no significant differences based on group, infection status, or time for the remaining three alpha diversity metrics (data not shown). In JejM microbiota, observed features significantly differed based on group (*H* = 20.47, *p* = 0.03, Figure 3B), with observed features being lower in infected birds on days 7 (*H* = 5.33, *p* = 0.02) and 10 (*H* = 5.00, *p* = 0.03). Additionally, Faith PD significantly differed based on group (*H* = 19.31, *p* = 0.04, Figure 3C), with Faith PD being lower in infected birds on day 7 (*H* = 4.08, *p* = 0.04) and tending to be lower on day 10 (*H* = 3.76, *p* = 0.053). Lastly, evenness significantly differed based on group (*H* = 19.07, *p* = 0.04, Figure 3D), with evenness being higher in infected birds on days 7 (*H* = 4.08, *p* = 0.04) and 14 (*H* = 5.33, *p* = 0.02). There were no significant differences in Shannon diversity based on group, infection status, or time (data not shown).

### 3.3. Beta diversity

**Figure 4:**
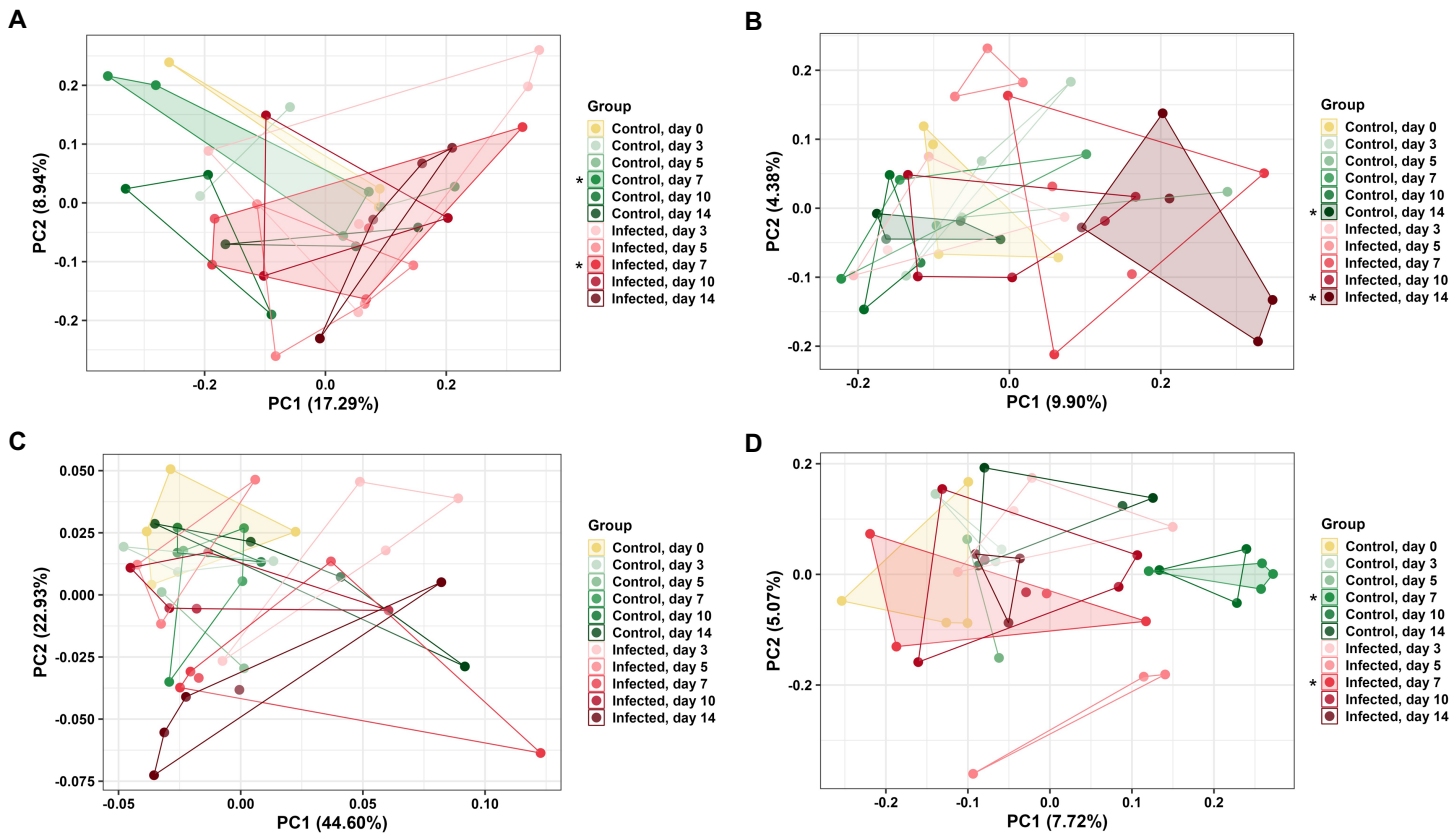
Principal coordinate analysis (PCoA) based on: **(A)** unweighted UniFrac distance matrix of duodenal luminal (DuoL) microbiota, **(B)** unweighted UniFrac and **(C)** weighted UniFrac distance matrices of duodenal mucosal (DuoM) microbiota, and **(D)** unweighted UniFrac distance matrix of jejunal mucosal microbiota (JejM). Stars (*) on legend denote microbiota were significantly different (PERMANOVA, *p* < 0.05) on those days. Longer distances between points indicate microbiota profiles were different, while shorter distances between points indicate profiles were similar.

DuoL microbiota were distinct based on group using the unweighted UniFrac distance metric (PERMANOVA, pseudo-*F* = 1.20, *p* = 0.04, Figure 4A), with the microbiota of infected birds differing from control birds on day 7 (pseudo-*F* = 1.60, *p* = 0.04). However, microbiota in different groups were similar using weighted UniFrac (*p* > 0.05). DuoM microbiota were distinct based on group using unweighted UniFrac (pseudo-*F* = 1.16, *p* < 0.01, Figure 4B), with the microbiota of infected birds differing from control birds on day 14 (pseudo-*F* = 1.82, *p* < 0.01). Groups of DuoM microbiota were also distinct using weighted UniFrac (pseudo-*F* = 1.57, *p* = 0.03, Figure 4C), though there was no significant difference on a particular day.

JejL microbiota were not distinct based on group using both unweighted and weighted UniFrac (*p* > 0.05, data not shown). JejM microbiota were distinct based on group using unweighted UniFrac (pseudo-*F* = 1.22, *p* < 0.01, Figure 4D), with the microbiota of infected birds differing from control birds on day 7 (pseudo-*F* = 1.59, *p* = 0.03). Using weighted UniFrac, there was no significant difference in microbiota between groups (*p* > 0.05, data not shown).

### 3.4. Differential bacterial abundance in infected and control birds

**Figure 5:**
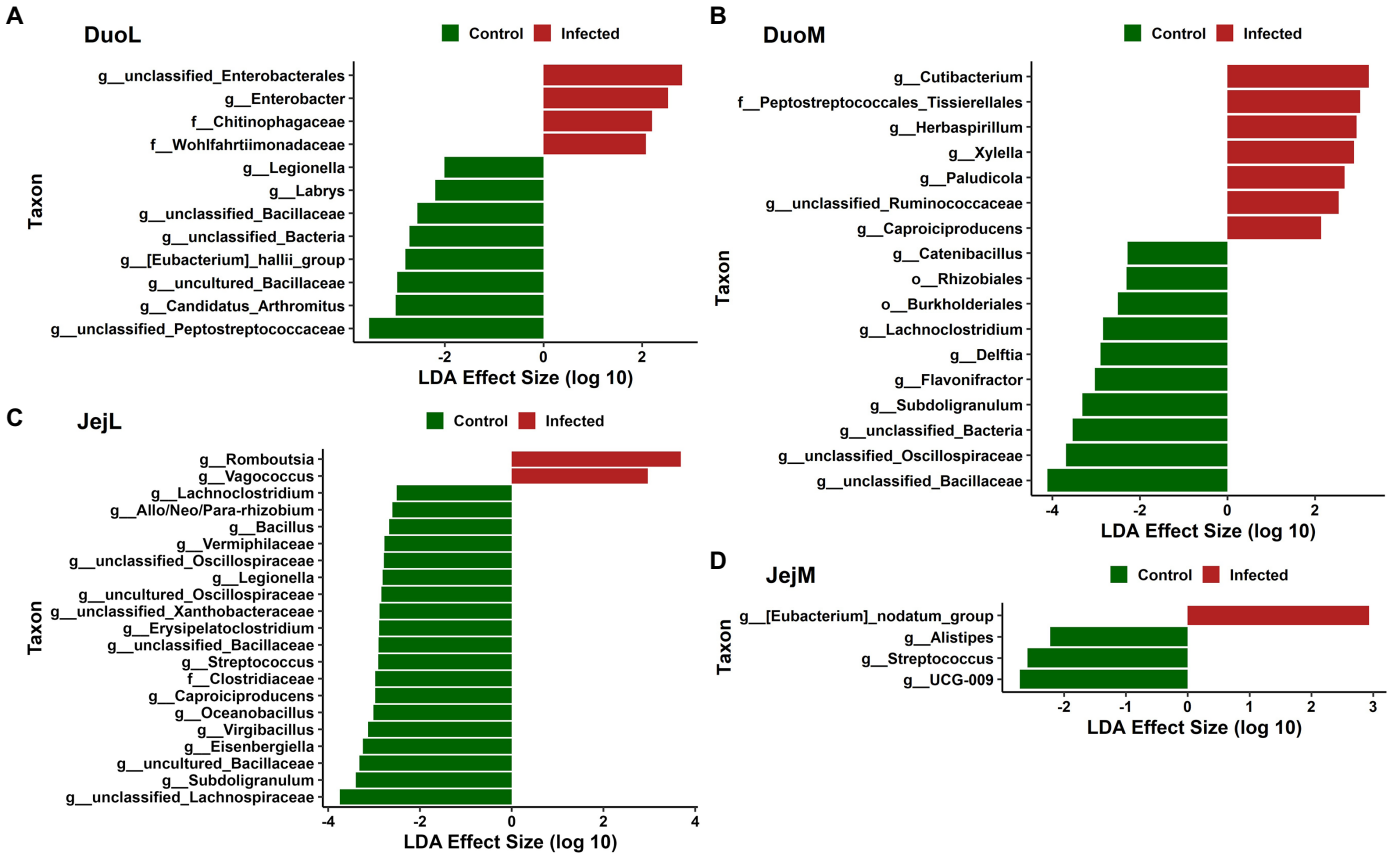
Linear discriminant analysis effect size (LEfSe) in: **(A)** duodenal luminal (DuoL) microbiota, **(B)** duodenal mucosal (DuoM) microbiota, **(C)** jejunal luminal (JejL) microbiota, and **(D)** jejunal mucosal (JejM) microbiota. Positive effect size indicates higher relative abundance in *Eimeria acervulina*-infected birds, while negative effect size indicates higher relative abundance in control birds.

In DuoL microbiota, two genera, unclassified Enterobacteriales and *Enterobacter*, and the families Chitinophagaceae and Wohlfahrtiimonadaceae, were greater in relative abundance in infected birds compared to control birds (Figure 5A). Eight genera, including unclassified Peptostreptococcaceae, *Candidatus Arthromitus*, uncultured Bacillaceae, and [*Eubacterium*] hallii group (brackets indicate contested names in SILVA) were greater in control birds (Figure 5A). In DuoM microbiota, six genera, including *Cutibacterium*, *Herbaspirillum*, and *Xylella*, and the family Peptostreptococcales-Tissierellales were greater in infected birds (Figure 5B). Eight genera, including unclassified Bacillaceae, unclassified Oscillospiraceae, and *Subdoligranulum*, and the orders Burkholderiales and Rhizobiales, were greater in control birds (Figure 5B).

In JejL microbiota, the genera *Romboutsia* and *Vagococcus* were greater in infected birds (Figure 5C). Eighteen genera, including unclassified Lachnospiraceae, *Subdoligranulum*, uncultured Bacillaceae, and *Eisenbergiella*, and the family Clostridiaceae were greater in control birds (Figure 5C). In JejM microbiota, [*Eubacterium*] nodatum group was greater in infected birds, and *UCG-009*, *Streptococcus*, and *Alistipes* were greater in control birds (Figure 5D).

### 3.5. Predicted functional abundances

**Figure 6:**
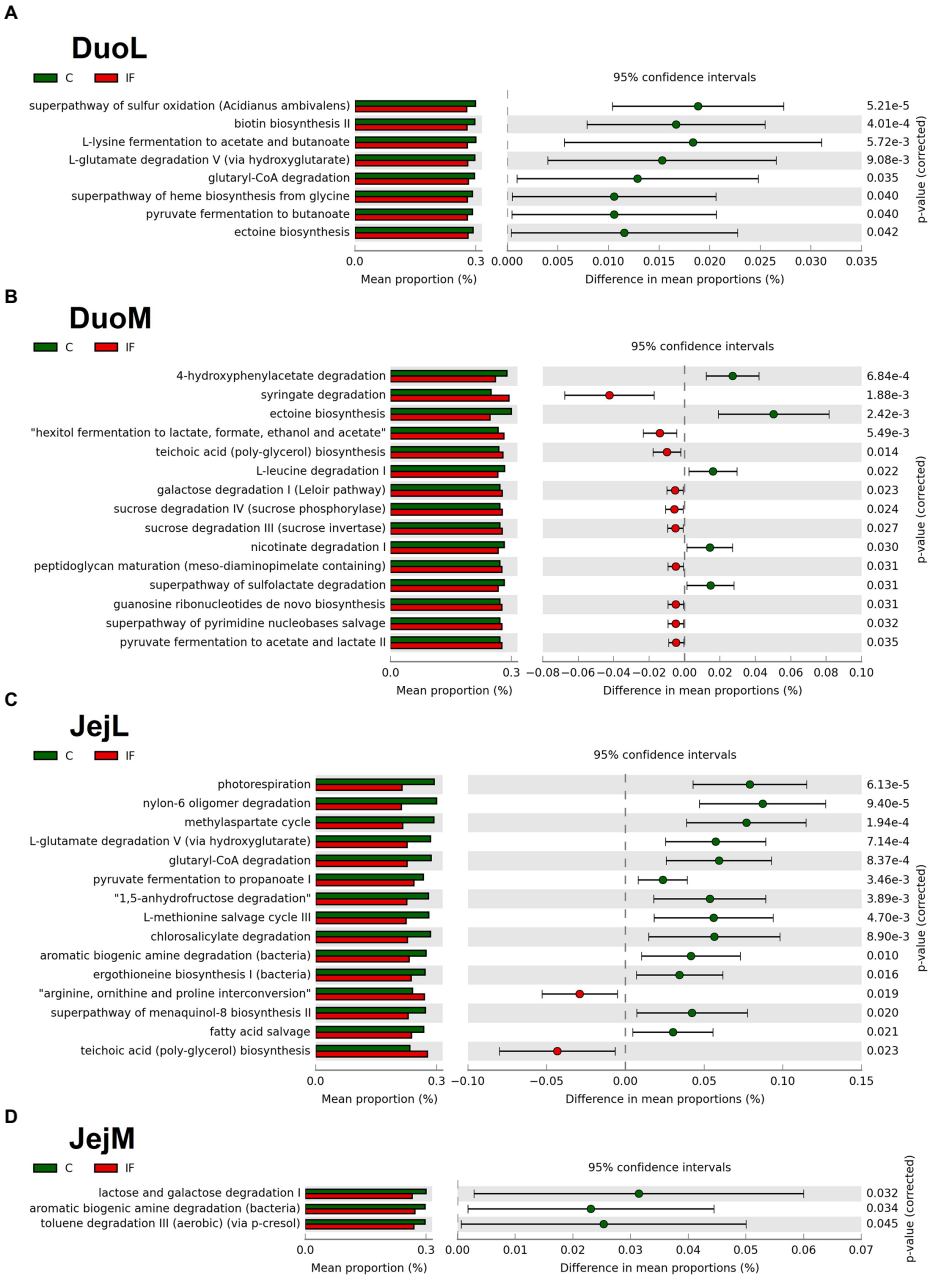
Effect of *Eimeria acervulina*-infection on mean proportion (%) of predicted MetaCyc pathways (up to the top 15 shown) in the **(A)** duodenal luminal (DuoL), **(B)** duodenal mucosal (DuoM), **(C)** jejunal luminal (JejL), and **(D)** jejunal mucosal (JejM) bacterial populations.

In DuoL microbiota, predicted metabolic pathways, including those for superpathway of sulfur oxidation, biotin biosynthesis II, L-lysine fermentation to acetate and butanoate, and L-glutamate degradation V (*via* hydroxyglutarate) were in greater abundance in C birds compared to IF birds (Figure 6A).

In DuoM microbiota, some predicted metabolic pathways such as 4-hydroxyphenylacetate degradation, ectoine biosynthesis, L-leucine degradation I, and nicotinate degradation I were in greater abundance in C birds, while other predicted pathways such as syringate degradation; hexitol fermentation to lactate, formate, ethanol, and acetate; teichoic acid (poly-glycerol) biosynthesis; and galactose degradation I (Leloir pathway) were in greater abundance in IF birds (Figure 6B).

In JejL microbiota, some predicted metabolic pathways such as photorespiration, nylon-6 oligomer degradation, methylaspartate cycle, and L-glutamate degradation V (*via* hydroxyglutarate) were in greater abundance in C birds, while other predicted pathways such as arginine, ornithine, and proline interconversion, teichoic acid (poly-glycerol) biosynthesis, mevalonate pathway I, and superpathway of glycolysis and Entner-Doudoroff were in greater abundance in IF birds (Figure 6C).

In JejM microbiota, predicted metabolic pathways including lactose and galactose degradation I, aromatic biogenic amine degradation, and toluene degradation III (aerobic) (*via* p-cresol) were in greater abundance in C birds compared to IF birds (Figure 6D).

## 4. Discussion

### 4.1. Luminal and mucosal microbiota profiles

This study focused on the effects of *E. acervulina* infection on the luminal and mucosal microbiota of the duodenum and jejunum, the primary sites of infection for *E. acervulina*. Luminal and mucosal microbiota have been shown to differ based on alpha and/or beta diversity in four regions of the GIT: the cecum, ileum, jejunum, and duodenum (Proszkowiec-Weglarz, 2022). Microbiota profiles of luminal and mucosal microbiota differed in both the duodenum and jejunum in our study, though the dominant taxa in the duodenum and jejunum did not greatly differ. Luminal microbiota in the duodenum and jejunum were typically dominated by *Lactobacillus*, with *Lactobacillus* often making up over 90% of the bacterial community. In samples where *Lactobacillus* percentage was below 95%, facultative anaerobes such as *Escherichia coli*, *Streptococcus*, and/or *Enterococcus* were often present, whose abundances may increase when oxygen availability is greater. Mucosal microbiota of the duodenum and jejunum were more varied, typically containing unclassified Bacillaceae, unclassified Lachnospiraceae, *Lactobacillus*, and various genera from the orders Lachnospirales, Oscillospirales, and Bacillales. These differences in luminal and mucosal microbiota demonstrate the need to understand the effects of infection in both types of microbiota, as different bacterial taxa in each microbiota may influence poultry health differently. Additionally, *Lactobacillus* strains are commonly tested as a probiotic to improve poultry health, and the differing abundances of *Lactobacillus* within the luminal and mucosal microbiota may provide insight to the role *Lactobacillus* plays in microbiota-infection interactions.

### 4.2. Effects of *Eimeria acervulina* infection on alpha diversity

Alpha diversity was affected by *E. acervulina* infection only in the mucosal microbiota of the duodenum and jejunum. In the duodenum, richness (based on phylogenetic diversity, but not observed features) was lower in infected birds on the last day of the study (day 14 PI, or birds 35 days of age), suggesting a possible long-term effect of infection on the phylogenetic diversity of DuoM microbiota, as this was past the peak infection period of 5 to 7 days PI for *E. acervulina* (Allen and Fetterer, 2002). Significantly reduced plasma carotenoids in IF birds on days 5 and 7 post-infection (Cloft et al., 2023) supported that this was the peak infection period in this study. JejM microbiota appeared to be affected differently, where both richness metrics (observed features and phylogenetic diversity) were found to be lower on days 7 and 10. This difference appeared to occur as control birds tended to see a sharp increase in richness between day 5 and day 7, while this increase did not occur in infected birds. A previous study has shown richness can increase in the jejunum in the first five weeks of development (Glendinning et al., 2019), and the peak infection period during 5–7 days PI suggests the infection prevented a normal increase in richness. Interestingly, evenness was higher in infected birds on days 7 and 14. Data from the control birds shows some decrease in evenness may be normal at day 7, suggesting the peak of infection may lead to a higher evenness than usual both at the peak of infection and one week PI. Given that richness was lower in infected birds at the peak of infection while evenness was higher, it is possible the absence of certain bacterial taxa promoted less dominance and a more even spread of bacterial taxa.

### 4.3. Effects of *Eimeria acervulina* infection on beta diversity

DuoL microbiota were distinct in infected birds compared to control birds on day 7 based on beta diversity, while DuoM microbiota were distinct on day 14. These results were driven by presence and absence of ASVs as considered by unweighted UniFrac, as there were no significant differences on a particular day with weighted UniFrac analyses, which consider the abundance of ASVs. Consistent with the observed long-term decrease in richness, this result supports that the DuoM microbiota may be affected in the long-term through presence and absence of certain bacteria. JejL microbiota were not distinct on any day while JejM microbiota were distinct on day 7, again showing the importance of distinguishing between luminal and mucosal microbiota. This result also suggests that JejM microbiota are affected more in the short term by *E. acervulina* infection compared to DuoM microbiota, despite the two regions sharing some similarities in bacterial taxa.

### 4.4. Differential abundance of bacteria from infection and potential links to short-chain fatty acid production

Differential abundance analysis revealed differences in relative abundance of certain taxa in IF chickens compared to control chickens. Cecal microbiota studies have suggested that *E. tenella* infection can lead to secondary outbreaks by opportunistic pathogens (Macdonald et al., 2017), and a previous study showed a large increase in *E. coli* relative abundance in IF birds along with smaller increases to *Enterococcus*, *Clostridium*, and *Proteus* (Campos et al., 2022b). The genus *Enterobacter*, known to contain pathogenic species, and unclassified Enterobacterales were taxa found in greater relative abundance in the DuoL microbiota of IF birds, while the periodontitis-causing [*Eubacterium*] *nodatum group* (Haffajee et al., 2006) was greater in the JejM microbiota of IF birds, with these taxa potentially acting as secondary pathogens. Interestingly, the antioxidant and anti-inflammatory feed supplement *Allium hookeri* can decrease the abundance of [*Eubacterium*] *nodatum group*, while the supplement’s overall changes to the gut microbiota are also correlated to improved body weight in chickens (Lee et al., 2020).

Another way *Eimeria* infection may impact poultry health through changes in the microbiota is by affecting abundances of bacteria producing short-chain fatty acids (SCFAs). SCFAs, especially butyrate, have been shown to improve body weight gain in chickens challenged with *E. maxima* (Proszkowiec-Weglarz et al., 2020; Hansen et al., 2021) and in chickens with nutrient-reduced diets (Bortoluzzi et al., 2017). Lower relative abundance of potential SCFA-producing bacteria in IF birds was a pattern in multiple microbiota in this study. This pattern was observed in DuoL microbiota with unclassified Peptostreptococcaceae, a family with butyrate-producing members in human infants (Appert et al., 2020). *Subdoligranulum* and *Flavonifractor*, of which there have been butyrate-producing species isolated from chicken cecum (Eeckhaut et al., 2011), followed the pattern in DuoM microbiota. In JejL microbiota, unclassified Lachnospiraceae, *Subdoligranulum*, and *Eisenbergiella* followed the pattern. Lachnospiraceae, known for production of SCFAs, has been shown to be in lower relative abundance in both the cecal luminal and mucosal microbiota of chickens infected with *E. tenella*, as well as being positively correlated with body weight gain (Campos et al., 2022b). *Eisenbergiella*, a genus within Lachnospiraceae, has isolates known to produce butyrate, lactate, acetate and succinate (Amir et al., 2014), and has been found to decrease in abundance in disturbed gut microbiota of rats (Luo et al., 2018). Though fewer differences were observed in JejM microbiota overall, *Alistipes*, a major succinate producer and minor propionate producer (Polansky et al., 2016; Parker et al., 2020), and *Streptococcus* followed the pattern. The species of *Streptococcus* in our study was unknown, however, it is notable that certain strains of *Streptococcus* isolated from chicken ceca in other studies have either shown butyrate production ability (Eeckhaut et al., 2011) or probiotic potential (Zhang et al., 2021). These results suggest that an *Eimeria* infection may affect abundances of SCFA-producing bacteria in regions of the gut other than the cecum.

### 4.5. Predicted functional abundances suggest potential effects from infection on short-chain fatty acid production pathways

Although it has been shown that bacterial abundances are affected by infection, less is known about the mechanisms affected by these changes in abundance. Analysis of predicted pathways using the MetaCyc database showed genes found in lower relative abundance in the DuoL microbiota of IF chickens included those predicted to influence pathways affecting acetyl-CoA production, such as L-lysine fermentation to acetate and butanoate (butyrate), L-glutamate degradation V (*via* hydroxyglutarate), glutaryl-CoA degradation, pyruvate fermentation to butanoate, and ectoine biosynthesis. Acetyl-CoA is utilized in two major pathways leading to butyrate production (Polansky et al., 2016), of which two of the listed pathways directly influence. Similar to DuoL microbiota, genes predicted to influence pathways affecting acetyl-CoA production were found in lower relative abundance in the JejL microbiota of IF chickens, with the methylasparate cycle pathway also among those significantly lower. Though the luminal microbiota of these locations tend to be dominated by *Lactobacillus*, the presence of bacteria known for expression of enzymes favoring butyrate production, such as *Subdoligranulum* and *Oscillibacter* (Polansky et al., 2016), may explain these results. Together with the differential abundance results, these findings suggest *E. acervulina* infection may influence butyrate production by affecting the microbiota, though the effects may be limited from the duodenum and jejunum when considering the exponentially higher bacterial density in the cecum. Further research on effects of *E. acervulina* infection on the cecal microbiota and correlation analyses between SCFA-producing bacteria and factors such as body weight gain may assist in determining which bacterial taxa have a greater influence on poultry health during infection.

### 4.6. Conclusion

*E. acervulina* infection led to changes in alpha diversity in only the mucosal microbiota, with seemingly longer-term effects in the duodenum while the jejunum was primarily affected in the shorter-term, and beta diversity results were consistent with this pattern. In general, relative abundance of potential SCFA-producing bacteria tended to be lower in *E. acervulina*-infected chickens in all four of the microbiota groups analyzed. Predicted metagenomic functions showed pathways related to SCFA production, especially butyrate, may be affected by these changes in relative abundance during infection. Further research on metagenomic function may provide insights on the degree of influence duodenal and jejunal bacteria have on chicken health.

## Data availability statement

The datasets presented in this study can be found in online repositories. The names of the repository/repositories and accession number(s) can be found at: NCBI – PRJNA892608.

## Ethics statement

The animal study was reviewed and approved by Institutional Animal Care and Use Committee (IACUC, protocol #18–025) of the Beltsville Agricultural Research Center.

## Author contributions

KM, MJ, and MP-W contributed to the conception, design, and investigation of the study. XY contributed to the initial statistical analysis and bioinformatics analysis. PC completed the final statistical analysis, bioinformatics analysis, visualization, and wrote the first draft of the manuscript. PC, KM, MJ, and MP-W contributed to manuscript revision. All authors have read and approved the submitted version of the manuscript.

## Funding

This research was funded by the in-house USDA-ARS CRIS project number 8042–31000-108-00D.

## Conflict of interest

The authors declare that the research was conducted in the absence of any commercial or financial relationships that could be construed as a potential conflict of interest.

The handling editor MK declared a shared affiliation with the authors at the time of review.

## Publisher’s note

All claims expressed in this article are solely those of the authors and do not necessarily represent those of their affiliated organizations, or those of the publisher, the editors and the reviewers. Any product that may be evaluated in this article, or claim that may be made by its manufacturer, is not guaranteed or endorsed by the publisher.
